# Some Hints on the Preparation and Mounting of Microscopic Objects

**Published:** 1882-06

**Authors:** W. H. Walmsley


### SOME HINTS ON THE PREPARATION AND MOUNTING OF MICROSCOPIC OBJECTS.

BY W. H. WALMSLEY.

THIRD PAPER.

It is a matter of regret, I think, to those who have taken the trouble to think at all about the matter, that in our microscopic work, the efforts of almost all observers and workers are mainly devoted to such preparations as require, at least, moderately high powers and carefully arranged transmitted light for proper showing. Doubtless this is the only method by which we can hope to obtain any knowledge of the real structures of tissues, animal or vegetable, or of inorganic substances, such as rocks; but there are thousands of objects in the limitless realms of nature which will afford instruction and delight to the more indifferent of observers, who view merely their exteriors with a low power. Many of those require neither preparation nor mounting, being too common or abundant to repay the slightest labor bestowed upon them. Others, however—and their name is legion—can and should be preserved in some permanent manner, readily accessible, and easily arranged for examination. In the vegetable kingdom we are presented with an endless and charming variety of beautiful forms in the seeds of even our commonest flowers, or even weeds, whilst the feathers, scales and hairs of the animal afford a never ending storehouse of treasures for the seeker after the curious and beautiful. The pollen from the tiniest flower, or the sands from the shores of the mighty ocean, alike present us forms and colors of surpassing beauty; and the preservation of these in a permanent form is at times most desirable. It shall be the purport of the present paper to point out some plain methods of doing so, which will produce good results if carefully followed.

Let us term this method of mounting "The Dry Way," to distinguish it from those preparations made in aqueous or other fluids, and proceed to make our mount in one of the several ways whereby it may be done. The books have been filled with such for years, good, bad, and indifferent. We have had full discussions of the merits and demerits of cells, possible and impossible; some made of shellac, turned upon a whirling table with the point of a pen-knife, at an immense expenditure of time and patience; others of wax, bone, tin, hard and soft rubber, curtain rings, and a host of other substances; anything, in fact, but those, or rather that possessing the one quality needful for a dry cell, namely, the quality of remaining dry. For be it distinctly understood that though a cell may be made and hermetically sealed, in which no appearance of moisture will ever occur, such an event is an anomaly and can never be duplicated with any certainty. No matter how dry the specimen may appear to be, nor the atmosphere of the room or the surface of the covering glass, sooner or later the under side of the latter will become covered by a mist like substance, which obscures and spoils the view of the imprisoned specimen. This of course is the case only with such preparations as are mounted on the bottom of a cell of any depth, the cover being used merely as a protection from dust and other injury. Where diatoms, the scales of insects, or other minute objects are mounted directly upon the under surface of the cover itself, this cloudy or watery appearance is either never observed at all, or else in so slight a degree as to cause no annoyances. When it does occur in a cell such as is usually used, and for the preparation of which the books give us so many elaborate directions, the only remedy is to remove it, (broken of course,) and replace with a fresh one; the latter in due time being predoomed to share a like fate.

"Is there no remedy for this?" you will ask, and I answer unhesitatingly yes, if you will sacrifice your artistic cells of wax or what not, with their pretty colored rings of varnish, and be content with those of humbler or far more useful qualities. Paper, of which such dissimilar articles are now manufactured, as love letters and car wheels, is our friend in need in this emergency. Not sized or glazed or calendered, but soft, porous paper of various thicknesses, to suit our needs; a thick blotting pad being exceedingly useful, for cells containing objects sufficiently thick to require such a depth. If a still deeper cell than this be needed, then a slip of wood, $3 \times 1$ inches, and the thickness of an ordinary glass slide, with a hole bored through the middle is most useful; and here again comes in our friendly paper to form the bottom, all of which will be dwelt upon in due course.

The requisites, then, for our dry mounting in the manner to be described, are as follows: Crown glass slips of the usual dimensions, $3 \times 1$, and of moderate thickness, with cut edges, (grooved or smooth ones are a needless expense), wooden slips of the same dimensions, with holes through their centers, $\frac{5}{8}$ to $\frac{3}{4}$ of an inch in diameter; covers of medium thickness, circles or squares as you prefer,--the latter being cheaper and equally good as the former; a supply of porous paper of various thicknesses from that of thick writing to a blotting board, two punches, $\frac{5}{8}$ and $\frac{3}{4}$ inches in diameter, some thin card board, covered with dead black paper, to form the bottom of the cells when the wooden slips are used, and a supply of colored paper for the backs and edges of the slides, with the bronzed or figured ones for the fronts of same. These latter may be purchased of any optician at a small outlay, and are made purposely in very neat and pretty patterns. Labels, of course, oval or round, as one fancies, and the tools and materials we have gathered together in our balsam mountings will suffice to give us a very pretty outfit wherewith to commence work on our dry mounts.

What shall we commence with? Here are some lovely little seeds, it may be of the portulaca, or the common chickenseed. Finding their diameter to be a little less than the thickness of our blotting pad, we will determine to make our cell of the latter, and so proceed to stamp a hole in a portion thereof with our $\frac{5}{8}$ inch punch, after which we cut out a square of $\frac{7}{8}$ inch, leaving the hole in the center. Before attaching this cell to the glass slip a dead black bottom must be made for it, and this is best done by pasting a strip of the thin black paper upon the slide. And here let me say that the best and most satisfactory paste for this and all subsequent processes I have ever used is made with ordinary wheat starch, boiled, and beaten to the consistency of thick cream. It adheres tenaciously to glass, wood, or paper, and seems to have no tendency whatever to absorb moisture from the surrounding atmosphere.

The black paper having been pasted upon the slide, the cell is in its turn to be pasted upon the paper so that its center shall be precisely in the center of the slide, when a weight should be placed upon it until dry, and finally attached. The seeds may be attached to the bottom of the cell by means of shellac cement, or liquid glue. Still better, and in every way satisfactory is a cement made by dissolving a small quantity of shred gelatine in cold water, gently heating it after being dissolved. This should be made in small quantities as wanted, since it will not keep. It is tough and very tenacious, and does not dry too quickly, excellent qualities in a cement for such purposes. Use only a sufficient quantity to attach the specimen firmly; any superfluity makes an unsightly blotch in the mount. The cleansed glass cover is now to be cemented on, with the same paste, when we are ready for the finishing.

The best colors for the covering papers are a bright canary for the back, and a red with gold bronze figures for the front, and these are the kind usually found on sale at the opticians.* The back should be pasted on the under side of the glass slip and turn up over the sides and ends on to the upper side of the same, over which it should extend for $\frac{1}{8}$ of an inch all around. Then the red and gold front with a $\frac{5}{8}$ inch hole previously punched in its center is to be pasted smoothly over the whole, equi-distant from the edges all around. The labels,—usually plain white ovals,—are to be placed upon each end, when the appearance of the whole mount will be extremely neat and handsome, and the maker's mind need bear no troubles as to its future. Should any appearance of moisture under the cover be seen—which is not at all likely—a slight warming over the lamp will dispel the same, and leave all in pristine brightness.

Should our specimen be too bulky or thick to be contained within the shallow depths of a cell made of the thinnest blotting pad, we must have recourse to the wooden slips, which will be found to form a cell deep enough for any mounting one may ever desire to make. The method of so doing is precisely the same as that followed with the glass slip, excepting that we must paste a strip of cardboard, covered with the dead black paper, to the under side of the slip to form the bottom of the cell. The slide is to be covered with the papers, and labeled exactly the same as though it were of glass.

Should the black paper not be readily procurable, a very excellent dead black for the bottom of the cell may be made with common lampblack water color, which dries with a dead surface very agreeable and pleasant for mounting foraminifera and similar objects upon. If a little gum arabic be mixed with the water, and the specimen placed upon the surface of the paint whilst still moist, it will be found that the latter will form an excellent cement, as well as background for holding the preparation.

Illumination by means of a lieberkuhn, which throws the light directly down upon the object without shadows, has been

* The writer regrets that the illustrations intended for this article have not been finished by the engraver in season to appear with the letter press.

too much neglected. In England it is a very ordinary method of viewing the opaque objects, and a lieberkuhn is usually furnished with very object-glass from a three inch to a four-tenths. But it is different here, and I am quite sure that the great majority of our observers, professional and amateur, are totally unacquainted with its use. I think this is to be regretted, since they lose much, both in pleasure and instruction from its non-employment. Hoping that its use may become more general, I will give a hint or two as to the method of mounting a preparation, to be examined by this form of illumination.

The lieberkuhn is a concave speculum fitting over the mounting of the objective so that the front lens of the latter project through the middle of the lieberkuhn. Parallel rays of light are thrown upwards from the surface of the plane mirror, which being received upon the concave face of the lieberkuhn, are in turn reflected downwards upon the object under view. The foci of the objective and lieberkuhn being coincident, it follows that when the specimen under view is brought precisely into that of the former, its illumination by the latter is at the best. The central rays of light, however, coming immediately beneath the object, must be stopped out by some opaque background to insure the best effect. There are many modes of effecting this, and most of them involve the use of the various cells, which are hermetically sealed. Their having been described at length by many more capable writers, I shall confine myself to the one method whereby our porous paper medium may be employed. For this purpose we shall need no additional tools or materials, save a large punch, say $\frac{7}{8}$ inch, and some small circles of thin glass of $\frac{1}{4}$ to $\frac{3}{8}$ inch in diameter. Placing a glass slip upon the two tubes, we proceed to paint a disc, (very slightly larger than the circle of glass to be used), exactly in its centre. This is to be done with asphalte or Brunswick black, and the slide set aside for the same to harden, which it will do in an hour or two, or, if necessary, may be hastened by heating gently over the lamp or on the brass table. A second coating of the asphalte is now to be applied, and a circle of thin glass slightly warm is to be placed upon its surface with the forceps and gently pressed down to exclude air and cause perfect adhesion over its entire under surface. We have now a perfectly opaque stop, with a clean glass surface, into which the most delicate object cannot sink and be lost, as is always the case if mounted directly upon the surface of asphalte, without the intersection of the thin glass. Bear this carefully in mind, and always use the glass circle if you wish to insure your preparation against disappearences in a black sea of death with the first hot spell.

The subsequent proceedings are almost the same as those first described in the present paper. The cell is to be made with the large punch, so as to leave ample space for the rays of light from the mirror to pass between its inside edges and the central stop, upon which the specimen is to be mounted. If one thickness of the paper or blotting paper be not sufficient, a second or third may be pasted upon it, until the desired depth is reached. And, of course, the covering paper for the back must be punched to allow the light to pass up, and not be put on solid, as mounts for ordinary illuminations are. The object is best attached to the glass circle by means of the gelatine cement, and the slide is to be finished precisely the same as heretofore directed. And finally, all fears of moisture spoiling a beautiful preparation in the future may be dismissed as groundless.

Our work thus far has been confined to opaque objects requiring surface illumination, and it may be said that the great majority of all to be mounted in the dry way are of this class. But many, notably scales and hairs of insects, and plants, many diatoms, sections of pith, etc., are best viewed in the dry state and by transmitted light. Most of these may be mounted upon the cover, (the method of doing so in the case of diatoms, having been directed in a former paper), and thus may be mounted in an ordinary cement cell without fear of moisture. The most satisfactory cement for this purpose (and most others), in my experience, is the white zinc, when properly prepared. It dries quickly, has no tendency to run in, and makes a beautiful finish to a mount. It should be used as follows, and the same directions will apply to asphalt or Brunswick black, if those cements be preferred:

Let us suppose that a $\frac{5}{8}$ inch cover is to be used. Placing our glass slip upon the turn-table, we proceed to run a ring of cement about its centre, the outer diameter of which shall be slightly in excess of $\frac{5}{8}$ inch, the width of the ring being about $\frac{1}{3}$ of an inch. Their first coat must be allowed to harden thoroughly, as on this depends all future success of the mount. If the slightest softness is left, it will be sure to yield still further in hot weather, and by capillary attraction run in and spoil the slide. To insure against all possibility of failure, let the slide be set aside for at least 48 hours, or else be baked in an oven. When the cover is ready to be placed upon it, a fresh ring of the cement is to be run upon the top of the first, extreme care being taken not to let the fresh extend to the inner edge of the old cement, lest it run in by contact between the surfaces of slide and cover. The latter is then to be placed upon the ring center with the forceps, and slightly pressed down. A very thin coating of the cement is now to be applied around the edge, and allowed to harden, after which as many may be applied, with or without colored rings, as the taste of the worker may dictate.

I find that the limit of my paper is reached without having exhausted the subject of which it treats, and the further consideration thereof must be left to a future one. In my next paper, therefore, I shall hope to finish this, and, at least, make a commencement on mounting in fluids, a subject that I regard as far more important than either balsam or dry mounts, and one in which practical hints, the result of many years of experience, may prove of more value to the beginner than anything I may have heretofore offered.

It may not be amiss to say, in concluding the present paper, that I have lately, after a long series of experiments, succeeded in perfecting an attachment, applicable to any microscope, whereby negative enlargements of all objects not requiring a greater power than $\frac{1}{4}$ of an inch, may be readily and perfectly made by any one, even totally unacquainted with photography, and from these positions printed for throwing upon the screen with a lantern ; at an infinitesimal cost of money and time. The whole process is performed by the simple aid of an ordinary coal oil lamp, neither Heliostat or any other costly form of illumination being required. I shall hope to describe and illustrate this apparatus in an early number of this magazine.—*The Microscope.*

